# Mechanical Design and Kinematic Modeling of a Cable-Driven Arm Exoskeleton Incorporating Inaccurate Human Limb Anthropomorphic Parameters

**DOI:** 10.3390/s19204461

**Published:** 2019-10-15

**Authors:** Weihai Chen, Zhongyi Li, Xiang Cui, Jianbin Zhang, Shaoping Bai

**Affiliations:** 1 The School of Automation Science and Electrical Engineering, Beihang University, Beijing 100191, China; whchenbuaa@126.com; 2 Beijing Machine and Equipment Institute, Beijing 100191, China; 3 The School of Mechanical Engineering and Automation, Beihang University, Beijing 100191, China;; 4 The Department of Materials and Production, Aalborg University, 9220 Aalborg, Denmark; shb@mp.aau.dk

**Keywords:** cable-driven exoskeleton, rehabilitation robot, upper limb

## Abstract

Compared with conventional exoskeletons with rigid links, cable-driven upper-limb exoskeletons are light weight and have simple structures. However, cable-driven exoskeletons rely heavily on the human skeletal system for support. Kinematic modeling and control thus becomes very challenging due to inaccurate anthropomorphic parameters and flexible attachments. In this paper, the mechanical design of a cable-driven arm rehabilitation exoskeleton is proposed to accommodate human limbs of different sizes and shapes. A novel arm cuff able to adapt to the contours of human upper limbs is designed. This has given rise to an exoskeleton which reduces the uncertainties caused by instabilities between the exoskeleton and the human arm. A kinematic model of the exoskeleton is further developed by considering the inaccuracies of human-arm skeleton kinematics and attachment errors of the exoskeleton. A parameter identification method is used to improve the accuracy of the kinematic model. The developed kinematic model is finally tested with a primary experiment with an exoskeleton prototype.

## 1. Introduction

Robot-assisted motion training for stroke patients is being widely applied in physical therapies; the approach has several advantages over the traditional motion training which is conducted by therapists. A rehabilitation robot can offer intensive and repetitive, long-duration motion training. An exoskeleton for upper-limb rehabilitation can be worn on the human arm and can provide the required torque on human arm joints for motion training. For these reasons, this approach has received a great amount of attention in recent years.

Based on their configurations, exoskeletons can be divided into two categories: serial [1,2,3] and parallel [4,5,6]. In serial exoskeletons, the actuators are mounted onto the joints of serial linkages. This requires alignment between the exoskeleton joints and human joints for motion training safety [7,8,9]. To overcome this limitation, some mechanisms which can realize joint alignment were designed in [10,11,12,13,14,15]. The ASSISTON utilizes a 3-RRP parallel mechanism, a Schmidt coupler, and so on to achieve joint axes adjustments [11,12,13]. In [14], Thalagala et al. designed a six-degrees-of-freedom (6-DOFs) shoulder joint mechanism which allows the user to move his/her shoulder joint center in the frontal and transverse planes. However, as redundant joints are required to avoid misalignments between the joint center of the upper limb and the exoskeleton joints, most alignment-free exoskeletons are complex and bulky, which is still a concern associated with serial exoskeletons. 

In the parallel type, the actuators of the exoskeleton are used to drive the parallel linkages. Exoskeletons of this type are able to passively adapt to the biological structure of human upper limbs. Some exoskeletons with parallel structures can be found in [16,17,18,19,20,21,22,23,24,25]. Among them, cable-driven exoskeletons use cables to transmit motion and forces. The first design of a cable-driven exoskeleton by Yang et al. [16] can be dated to 2004. It uses ten cables to actuate the exoskeleton and yields 7-DOFs motion ability for upper limb. Recently a number of cable-driven exoskeletons have been developed for clinical rehabilitation, which can be found in [17,18,19,20,21]. Compared with conventional rigid-linkage exoskeletons, cable-driven exoskeletons have simple and light weight structures, and are able to avoid misalignments between the joint center of the upper limb and the exoskeleton joints. However, as cable-driven exoskeletons utilize the human upper limb as the mechanical structure, the kinematic parameters of upper limbs play a significant role in the kinematics of the human-robot system. This brings uncertainties in kinematics due to different upper limb sizes. Moreover, the attachment of the exoskeleton to the upper limb is flexible, which also brings uncertainties. In using such an exoskeleton in physical therapies, kinematic uncertainties must be reduced to improve the robustness and accuracy of the motion control.

In this paper, a cable-driven arm rehabilitation exoskeleton with a custom-designed arm cuff is presented. The arm cuff is designed with a two-stage structure, which attempts to improve the stability and comfort of the physical human–robot interaction. The kinematic model of the exoskeleton is established, and a parameter identification method is used to improve the accuracy of the model by reducing uncertainties from human-arm skeleton kinematics and the attachment of the exoskeleton to the upper limb. Experiments with the new exoskeleton and motion capture system were performed to evaluate the kinematic uncertainties and demonstrate the improvement of this newly-designed kinematic model.

The reminder of the paper is organized as follows. A biomechanical model of the human upper-limb is given in Section 2. The mechanical design of the cable-driven arm rehabilitation exoskeleton is presented in Section 3. The kinematic modeling, error analysis, and uncertainty identification method are described in Section 4. In Section 5, experiments are carried out to evaluate the effectiveness of the new design in improving the accuracy of kinematics. Section 6 concludes this paper.

## 2. Biological Structure of the Upper-Limb

As the cable-driven exoskeleton becomes integrated with the upper limb to become fully constrained, an irregularity analysis of the arm structure is necessary for the mechanism design and for the modeling of the exoskeleton. 

### 2.1. The Irregularity of the Upper-Limb Kinematics

The arm structure consists of three segments, the upper-arm, the forearm, and the hand, which are interconnected by skeletal joints, i.e., the shoulder, elbow, and wrist joints. This paper focuses on designing an exoskeleton for shoulder and elbow motion assistance. The shoulder joint (ball in socket joint) enables shoulder flexions/extensions, abductions/adductions, and inward/outward rotations. The elbow joint enables 1-DOF elbow flexions/extensions. The rotation axis of the arm skeletal joint moves as the arm moves [26]. The lengths of the limb segments change based on the arm anatomy of the limb.

### 2.2. The Irregularity of the Upper-Limb Contour

The upper-limb contour is mostly determined by the shape of the arm skeleton, muscles, and skin. The specific surface contour causes arm structure irregularities to occur too. Irregular arm contours can be described in two directions. In the longitudinal direction, the arm central axis is not straight due to the arm bones; this leads to misalignments between the human limb and the exoskeleton joints axes. In the circumferential direction, the arm has an irregular surface due to the presence of muscles. The arm transverses are not circular or elliptical, but have all kinds of dimensions. Therefore, a wearable exoskeleton should have the capacity to adjust to these variable dimensions.

## 3. Mechanical Design

**Figure 1:**
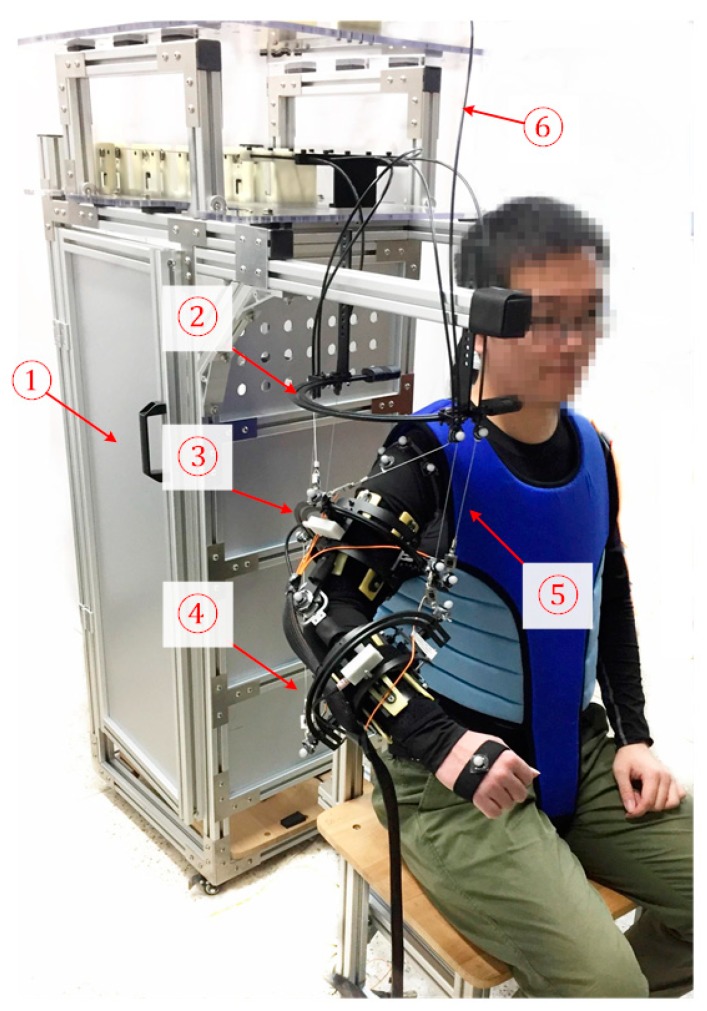
Cable-driven exoskeleton system constructed with (1) base frame, (2) base cuff, (3) upper-arm cuff, (4) forearm cuff, (5) cable, (6) Bowden cable.

The cable-driven arm rehabilitation exoskeleton system is shown in Figure 1. As can be seen, the user is sitting in a chair while wearing the exoskeleton. A binding vest fixing the subject body to the chair was used in an attempt to increase the stability of the motion training. The exoskeleton is mounted onto the user’s upper limb through the use of three cuffs, namely, a base cuff, upper-arm cuff, and forearm cuff, which allow to provide high force and torques in rehabilitation motion training. 

**Figure 2:**
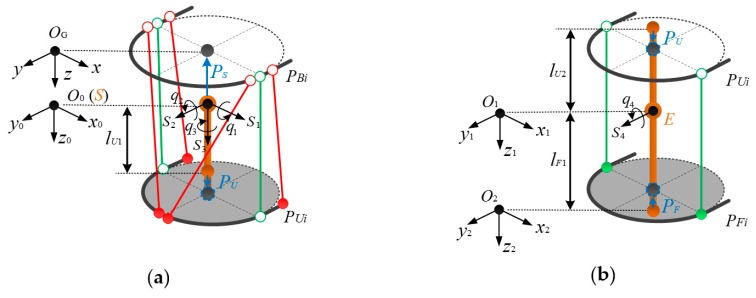
Schematic diagram of (**a**) the shoulder and (**b**) the elbow modules. In the diagram, local frames, $\left\{O_{0}\right\}$, $\left\{O_{1}\right\}$, and $\left\{O_{2}\right\}$ are located on the center of the shoulder joint, the center of the elbow joint, and the attachment point between the forearm cuff and human forearm, respectively; the global frame $\left\{O_{G}\right\}$ is located at the center point of base cuff.

The exoskeleton shown in Figure 1 is used for motion training of the shoulder and elbow joints. The exoskeleton can thus be divided into two independent modules, e.g., 3-DOFs shoulder module and 1-DOF elbow module, as shown in Figure 2. The 3-DOFs shoulder module shown in Figure 2a consists of base cuff and an upper-arm cuff. The base cuff is mounted onto the base frame, while the upper-arm cuff is fastened to the human upper-arm. In the shoulder module, the upper-arm cuff can be considered as a moving platform [27,28,29,30] which rotates around the human shoulder joint. The 1-DOF elbow module shown in Figure 2b consists of an upper-arm and a forearm cuff. The forearm cuff is fastened to the human forearm. The forearm cuff is the moving platform which can rotate around the elbow joint. In the exoskeleton, six cables are routed from DC motors (model: Maxon RE35) mounted on the based frame to the upper-arm and forearm cuffs through Bowden cables

**Figure 3:**
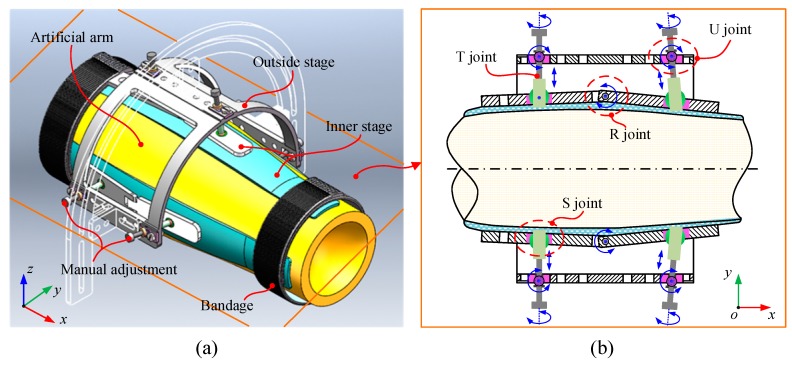
Design of the custom-designed cuff. (**a**) CAD model, and (**b**) projection drawing on the x-y plane.

In the exoskeleton, the design of the cuffs was developed based on the requirements of human comfort and the stability of the exoskeleton structure. Figure 3 shows the CAD model of the cuff. As shown in the figure, the cuff has a two-stage structure. The inner stage is made of a flexible silicon shell and can be fitted closely to the arm skin. The inner stage with flexible material can uniformly distribute the preload and pulling force on the arm skin surface generated from the cable tensions. The outside stage is designed with a rigid aluminum frame which can provide connecting points for the cables. Ensuring a firm attachment between the cuff and the human arm segment is important for the design of the cuff. In the cuff design, three sets of parallel mechanisms are applied to connect the inner and outside stages together. The parallel mechanism for exoskeleton assembly adjustment is designed based on an analysis of the irregular contours of the arm. It is intended to decrease the number of misalignments between the exoskeleton and the arm through adjusting the central axis of the exoskeleton to coincide with the arm. A schematic diagram of the parallel mechanism is shown in Figure 3b. The mechanism consists of three two-leg chain which can adjust assembly errors in their direction. Each two-leg chain has a configuration of U (universal joint), T (translational joint), S (spherical joint), and R (rotational joint), and can also be regarded as a parallel chain that consists of two U-T-S chains connected together by the R joint. Basically, the T joint is actuated manually to adjust the error in the circumferential direction and the R joint serves to fit the arm structure irregularity passively in the longitudinal direction.

## 4. Kinematic Modeling and Identification

### 4.1. Kinematic Modeling

A kinematics analysis of the exoskeleton system can be divided into two parts, i.e., the kinematics of the human arm skeleton and the kinematics of the cable-driven modules.

(1) *Kinematics of human arm skeleton*: The arm skeleton can be regarded as a serial open-chain mechanism. In Figure 2, the screw coordinates of the four arm joints in the global frame $\left\{O_{G}\right\}$ are denoted as $s_{i}\in R^{6\times 1}\;\left(i=1,\cdots ,4\right)$. The joint rotations are denoted as $q_{i}$. According to the POE formula, the nominal end pose $g_{O_{G}O_{2}}$ can be obtained as:
  (1) $$g_{O_{G}O_{2}}\left(q_{1},\cdots ,\;q_{4}\right)=\exp\left(\overset{3}{\underset{i=1}{\sum }}{\hat{s}}_{i}q_{i}\right)=\exp\left({\hat{s}}_{4}q_{4}\right)\cdot T_{O_{G}O_{2}}\left(0\right)$$
  It should be noted that the irregular structure of the upper limb joints gives rise to position deviations of screw coordinates $s_{i}\in R^{6\times 1}\;\left(i=1,\cdots ,4\right)$. Herein, the deviation between the frame $\left\{O_{G}\right\}$ and frame $\left\{O_{0}\right\}$ caused by shoulder joint center motion is denoted as $\delta P_{S}$, while that between the frame $\left\{O_{0}\right\}$ and frame $\left\{O_{1}\right\}$ caused by elbow joint center motion is denoted as $\delta P_{E}$. The effects of $\delta P_{S}$ and $\delta P_{E}$ on the homogeneous transformation matrices between the frames $\left\{O_{G}\right\},\;\left\{O_{0}\right\}$ and $\left\{O_{1}\right\}$ can be written as:
  (2a) $$\delta T_{O_{G}O_{0}}=\left[\begin{array}{cc} I & \delta P_{S}\\ 0 & 1 \end{array}\right]$$
  (2b) $$\delta T_{O_{0}O_{1}}=\left[\begin{array}{cc} I & \delta P_{E}\\ 0 & 1 \end{array}\right]$$
  Taking Equation (2) into consideration, Equation (1) is modified as
  (3) $$g_{O_{G}O_{2}}\left(q_{1},\cdots ,\;q_{4}\right)=\left[\begin{array}{cc} I & \delta P_{S}\\ 0 & 1 \end{array}\right]\cdot \exp\left(\overset{3}{\underset{i=1}{\sum }}{\hat{s}}_{i}q_{i}\right)\cdot \left[\begin{array}{cc} I & \delta P_{E}\\ 0 & 1 \end{array}\right]\cdot \exp\left({\hat{s}}_{4}q_{4}\right)\cdot T_{O_{G}O_{2}}\left(0\right)$$
(2) *Kinematics of cable-driven modules*: In Figure 2, the shoulder and elbow modules can be regarded as cable-driven parallel platforms, in which the moving platforms rotates around the shoulder and elbow joints relative to the base platform. Based on the cable-routing structure, the kinematics of the human arm skeleton and the attachment positions of the exoskeleton on the upper limb, the kinematic relationship between the human arm joint rotations and the motor outputs can be determined.

In the shoulder module, the cable lengths are obtained as:

(4) lis=‖P O0BiP O0Ui→‖=‖R O0O1P O1Ui−P O0Bi‖,  (i=1,⋯,m)

where P O0Bi=[P O0Bxi;P O0Byi;P O0Bzi;]. denotes the positions of the cable-routing points of the base cuff in frame $\left\{O_{0}\right\}$, P O1Ui=[P O1Uxi;P O1Uyi;P O1Uzi;] denotes the positions of cable-routing points of the upper-arm cuff in frame $\left\{O_{1}\right\}$, and R O0O1 is the rotation matrix from frame $\left\{O_{1}\right\}$ to frame $\left\{O_{0}\right\}$. 

With this in mind, the numbers of cables used in the shoulder and elbow modules are represented by *m* and *n*.

The cable lengths in the elbow module are obtained as:

(5) liE=‖P O0UiP O0Fi→‖=‖R O0O2P O2Fi+R O0O1PE−R O0O1P O1Ui‖ (i=m−n+1,…, m)

where P O2Fi=[P O2Fxi;P O2Fyi;P O2Fzi;] denotes the positions of the cable-routing points of the forearm cuff in frame $\left\{O_{2}\right\}$ and R O0O2 is the rotation matrix from frame $\left\{O_{2}\right\}$ to frame $\left\{O_{0}\right\}$.

In Equations (4) and (5), P O0Bi, P O1Ui, and P O2Fi change as the exoskeleton moves, due to the floating shoulder joint center and flexible attachment of the exoskeleton to the upper limb. Taking the motion of shoulder joint center $P_{s}$ and attachment errors $P_{U}$ and $P_{F}$ of the upper-arm and forearm cuffs into consideration, the positions of the cable routing points are modified as:

(6) {P O0Bi=P˜ O0Bi+Ps (i=1,⋯,m)     P O1Ui=P˜ O1Ui+PU (i=1,⋯,m)P O2Fi=P˜ O2Fi+PF (i=m−n+1,⋯,m)

where P˜ O0Bi, P˜ O1Ui, and P˜ O2Fi are the nominal attachment positions in the base, upper-arm, and forearm cuffs, respectively.

The lengths of the cables in the shoulder and elbow modules, $l_{i}^{s},i=1,\cdots ,m$ and $l_{i}^{E},\;i=m-n+1,\ldots ,\;m$, can be obtained using the inputs of the motor rotations, $\theta_{i},\;i=1,\cdots ,m$, which are given by:

(7) {lis=l 0iS+riθi  (i=1,⋯,m−n)liE=l 0iS+l 0iE+riθi  (i=m−n+1,⋯,m)

where l 0iS and l 0iE represent the initial cable lengths in the shoulder and elbow modules, respectively, and $r_{i},\;i=1,\cdots ,m$ are the radii of winches which are directly installed along the motors.

Based on Equations (2), (3), and (5), the kinematics of the shoulder and elbow modules can be determined as:

(8) {fiS=(l 0iS+riθi)2−(lis)2=0, (i=1,⋯,m−n)              fiE=(l 0iE+l 0iS+riθi−liS)2−(liE)2=0, (i=m−n+1,⋯,m)

Expressions of ${\left(l_{i}^{s}\right)}^{2}$ and ${\left(l_{i}^{E}\right)}^{2}$ are included in Appendix A.

### 4.2. Kinematic Identification

In the kinematic model, uncertain parameters have effects on the accuracy and robustness of the model. In this paper, a model based method which was used in our previous works [20,21] helped us to identify these uncertain parameters. 

In the kinematics Equations (8), the uncertain parameters are the following: $P_{U}$, $P_{F}$, $P_{S}$, $P_{E}$, l 0iS, and l 0iE. The identification model can thus be obtained by differentiating Equation (6) about uncertain parameters and the inputs $\theta_{i}$ of exoskeleton system, as follows:

(9) ∂fi∂θi·δθi+∂fi∂l 0i·δl 0i+∂fi∂P·δP+∂fi∂O·δO=0

where $P=\left[P_{S};P_{E}\right]$, $O=\left[P_{U};P_{F}\right]$, l 0i=[l 0iS;l 0iE].

Expressing Equation (9) in the matrix form, we get

(10) $$A_{m\times m}\underset{\delta X}{\cdot \underbrace{{\left[\begin{array}{c} \delta X_{S}\\ \delta X_{E} \end{array}\right]}_{m\times 1}}}=\underset{J}{\underbrace{{\left[\begin{array}{cc} J_{S} & 0\\ 0 & J_{E} \end{array}\right]}_{m\times \left(m+12\right)}}}\underset{\delta Y}{\cdot \underbrace{{\left[\begin{array}{c} \delta Y_{S}\\ \delta Y_{E} \end{array}\right]}_{\left(m+12\right)\times 1}}}$$

with

{δXS=[δθ1;δθ2;⋯;δθm−n]∈R(m−n)×1δXE=[δθm−n+1;⋯;δθm]∈Rn×1         δYS=[[δl 01S;⋯;δl 0m−nS];δPS;δPU]∈R[m−n+6]×1   δYE=[[δl 0m−n+1E;⋯;δl 0mE];δPE;δPF]∈R[n+6]×1A=−diag[∂f1S∂θ1,⋯,∂fm−nS∂θm−n,∂fm−n+1E∂θm−n+1⋯,∂fmE∂θm,]∈Rm×m JS=[diag(∂f1S∂l 01S,⋯,∂fm−nS∂l 0m−nS),[∂f1S∂PS;⋯;∂fm−nS∂PS],[∂f1S∂PU;⋯;∂fm−nS∂PU]]∈R(m−n)×[m−n+6]JE=[diag(∂fm−n+1E∂l 0m−n+1E,⋯,∂fmE∂l 0mE),[∂fm−n+1E∂PE;⋯;∂fmE∂PE],[∂fm−n+1E∂PF;⋯;∂fmE∂PF]]∈Rn×[n+6]

The least-square solution of the Equation (10) about δY is

(11) δY=pinv(J)AδX

where pinv(J) is the pseudo-inverse of J.

**Figure 4:**
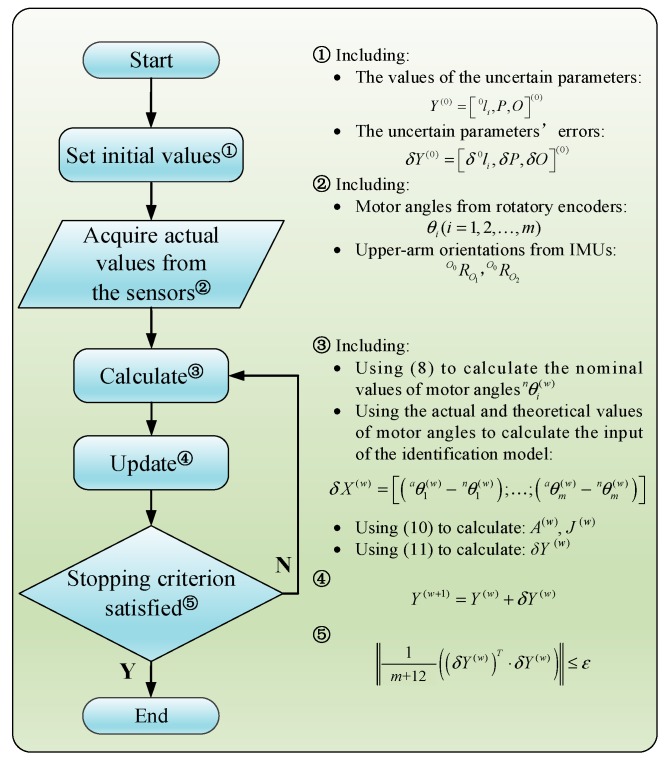
Flowchart of the identification of uncertain parameters.

Finally, the iteration method for the identification of uncertain parameters shown in Figure 4 is used to find solutions for the identification model. In this method, the deviations, δX, between the nominal rotation and real rotation angles of the motors are the inputs, while the deviations of uncertain parameters δY between *w* and *w*+1 iteration steps are defined as the outputs. As shown in Figure 4, the iteration is repeated until the output satisfies the stopping criterion

(12) $$\left|\frac{1}{m+12}\left({\left(\delta Y^{\left(w\right)}\right)}^{T}\cdot \delta Y^{\left(w\right)}\right)\right|\leq \varepsilon$$

The estimated values of uncertain parameters are updated using

(13) $$Y^{\left(w+1\right)}=Y^{\left(w\right)}+\delta Y^{\left(w\right)}$$

With the estimated values, the kinematic model of the exoskeleton can be updated.

Note that the identification model contains m equations but has m+12 unknown parameters. To find the solution for the identification model, at least num≥(m+12)/m sets of data from the sensors are required.

A simulation model of the exoskeleton was developed in MATLAB to test the identification method. In the simulation, the rotation angles $\theta =\left[\theta_{1},\ldots ,\theta_{6}\right]$ of the motors and limb orientations R=[R O0O1,R O0O2] were acquired from the simulation model in MATLAB. Ten sets of data of θ and R were used in the simulation. The stopping criterion ε was set as 0.1.

**Figure 5:**
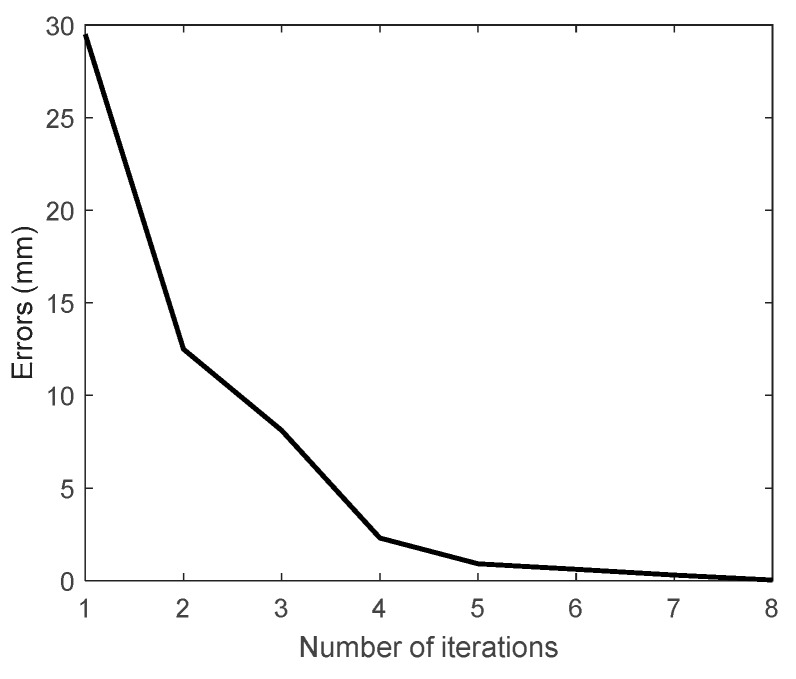
Iteration error versus number of iterations in the simulation.

The simulation results are depicted in Figure 5. We can see that after 8 iterations, the error was smaller than the defined value of the stopping criterion, demonstrating the convergence of the identification method. The elapsed time in MATLAB was 0.129 s. We can further use the identification method in our experimental study.

## 5. Experiments

**Figure 6:**
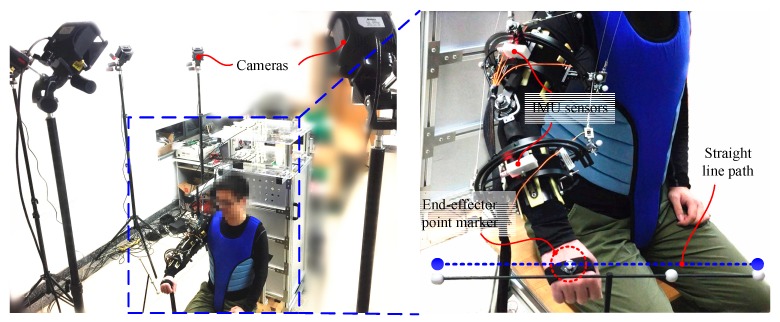
Experimental setup described as a subject tracks a line path. The motion of the subject’s upper limb is characterized by both IMU sensors placed on the exoskeleton and a motion capture system.

In this section, the effectiveness of the method of identification of uncertain parameters in a practical application is verified through experiments on a cable-driven exoskeleton prototype, as shown in Figure 6.

### 5.1. Experiment Setup

In the exoskeleton system, rotations $\theta =\left[\theta_{1},\ldots ,\theta_{6}\right]$ of the motors were acquired using encoders (model: HEDL-5540) which are connected in series with the motors. Two inertial measuring units (model: HiPUNC HI21X) mounted on the upper-arm and forearm cuffs were used to measure the orientations R=[R O0O1,R O0O2] of the upper and forearm limbs. θ and R, acquired from the exoskeleton system in the real world, are used as the inputs for the identification of uncertain parameters. 

Two subjects, namely A and B, participated in the experiments. In the experiments, the subjects were asked to wear the exoskeleton and sit on a chair. The task was to move their right upper limbs to track a line path four times. The line path is shown in Figure 6. In the task, the exoskeleton system worked in the human-in-charge mode, in which the cables were controlled at a constant tension of 20 N. With load cells (model: Forsentek FS01-10kg), a PID force feedback controller was applied to control the motors in order to allow the cables to follow the desired tension. In the task, 500 sets of measurement data from the sensors of the exoskeleton system were acquired, and every 10 sets of data were used for the identification of uncertain parameters. Thus, 50 kinematic models of the system could be obtained.

To verify the accuracy of the kinematic models with identified parameters, a Nokov motion capture system was used, as shown in Figure 6. In the motion capture system, a marker was mounted onto the hand of the subjects to measure their tracking results in the experiments. Fourteen markers were mounted onto the cable-routing points of the cuffs to capture changes in the lengths of the cables in the exoskeleton in the tracking task. A marker mounted on the subject’s shoulder joint recorded the movement of the joint center. Noting that as the Nokov system captures motion with sub-millimeter accuracy, the measurements can be considered as the real results in this work. The captured results can be compared with the identified results to validate the accuracy of the kinematic model.

### 5.2. Experiment Results

**Figure 7:**
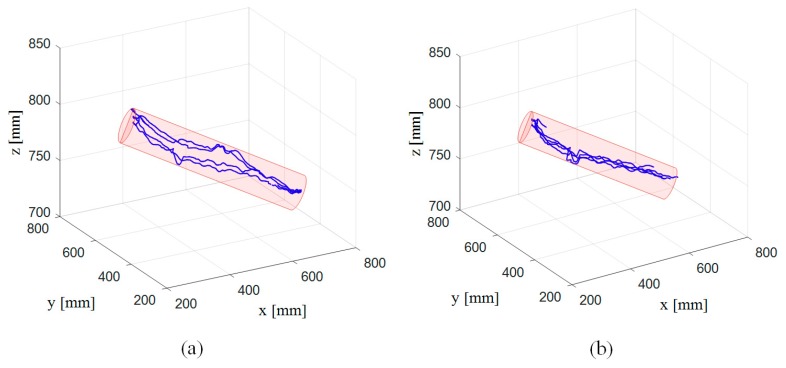
Tracking results of the experiments for (**a**) Subject A and (**b**) Subject B.

Figure 7 shows the tracking results of the subjects. As shown in the figure, the tracking results are close to the predefined trajectory. In the tracking task, the exoskeleton system runs stably, and the data acquired from the encoders and inertial measuring units can be used in the identification of uncertain parameters. Table 1 and Table 2 show the identified results for Subjects A and B, respectively. In the tables, the identified results are represented by the mean and root mean squared error (RMS error). As shown in Table 1 and Table 2, the uncertain parameters in the kinematic model fluctuate significantly as upper limb moves. Kinematic models with these identified parameters can be used to calculate changes of the cable length in the exoskeleton. The calculated results were further compared with the results acquired from motion capture system to validate the kinematic models.

**Figure 8:**
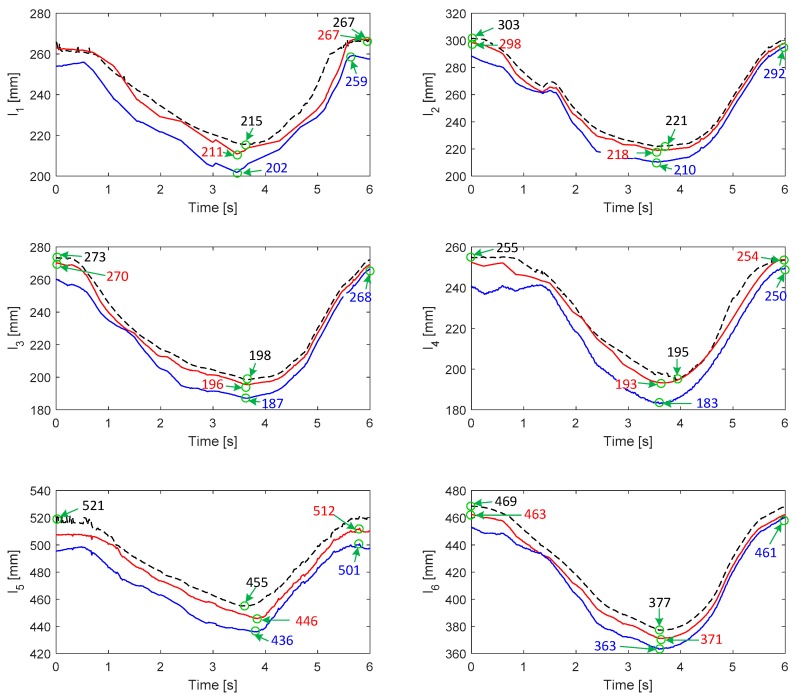
Changes of cables lengths in the experiment. The dotted lines denote the results obtained from the motion capture system. The red solid lines denote the calculated cables lengths based on the kinematic model with parameter identification; the blue solid lines denote the calculated results based on the kinematic model without parameter identification.

Figure 8 shows the changes of cables lengths in the 4th experiment for Subject A. As shown in the figure, the results calculated from the kinematic model with identification fit the results acquired from the motion capture system better than that without identification. The RMS errors between the captured results and the identified/unidentified results for both Subject A and Subject B are summarized in Table 3. The results indicate that the kinematic model can be improved effectively through the identification of uncertain parameters.

**Figure 9:**
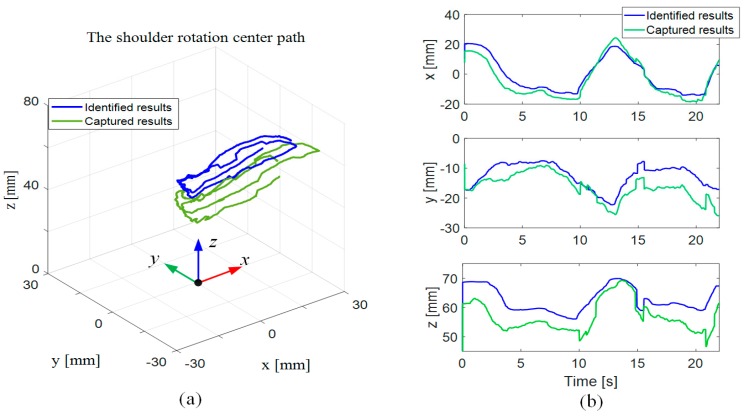
The movement of the shoulder joint center in (**a**) 3D and (**b**) 2D plots.

Figure 9 shows the variations of the position of the human shoulder joint center as the limb moves; the green solid curves denote the results measured from the motion capture system, and the blue curves denote the identified results of parameter $P_{S}$. As shown in the figure, the results of $P_{S}$ are generally in accordance with those measured from the motion capture system, showing a good predictive ability of the movement of the human shoulder joint. 

## 6. Conclusions

The major accomplishment of this paper is the mechanical design of a cable-driven arm rehabilitation exoskeleton with a new, custom-designed arm cuff. The new arm cuff shows good adaptability to the human arm, and can reduce uncertainties caused by instabilities between the exoskeleton and the human arm. Another major feature is the kinematic modelling of the exoskeleton. Uncertainties from the inaccuracy of human-arm skeleton kinematics and wearing errors of the exoskeleton are considered. An error model is built, with which an iteration method is used to identify uncertain parameters. A primary experiment on the prototype is carried out to demonstrate the improvements of the model. In future work, the improved kinematic model will be used in the motion control of the exoskeleton.

## Figures and Tables

**Table 1:** Identified results of uncertain parameters of the exoskeleton working with Subject A.

Parameters	Unidentified [mm]	Identified [mm]
$P_{S}$	[0; 0; 62]	[2 ± 10.4; 15 ± 4.5; 60 ± 5]
$P_{E}$	[0; 0; 280]	[0; 0; 274 ± 3.2]
$P_{U}$	[0; 0; 0]	[6 ± 4.1; 4 ± 3.3; 2 ± 2.2]
$P_{F}$	[0; 0; 0]	[1 ± 3.1; 8 ± 4.3; 3 ± 1.7]

**Table 2:** Identified results of uncertain parameters of the exoskeleton working with Subject B.

Parameters	Unidentified [mm]	Identified [mm]
$P_{S}$	[0; 0; 71]	[6 ± 5.9; 11 ± 8.7; 74 ± 5.1]
$P_{E}$	[0; 0; 273]	[0; 0; 278 ± 5.6]
$P_{U}$	[0; 0; 0]	[3 ± 5.2; 2 ± 3.4; 1 ± 1.9]
$P_{F}$	[0; 0; 0]	[2 ± 1.7; 3 ± 2.1; 4 ± 2.1]

**Table 3:** RMS errors of *l_i_* between captured results and calculated results based on identified/unidentified models.

Subject		RMS Error [mm]					
		$l_{1}$	$l_{2}$	$l_{3}$	$l_{4}$	$l_{5}$	$l_{6}$
**A**	Unidentified	11.9	10.8	10.8	12.3	19.5	13.1
	Identified	5.1	3.6	3.5	4.1	8.3	6.6
B	Unidentified	15.6	14.3	7.1	17.4	13.6	13.2
	Identified	4.4	7.8	3.4	2.5	6.3	7.1

## Appendix A

(A1) (liS)2=(‖R O0O1P O1Ui−P O0Bi‖)2=[P O1Uzi·(sq1·sq3+cq1·cq3·sq2)−P O1Uyi·(cq1·sq3+cq3·sq1·sq2)+P O1Uxi·cq2·cq3−P O0Bxi]2+[P O1Uyi·(cq1·cq3+sq1·sq2·sq3)−P O1Uzi(cq3·sq1−cq1·sq2·sq3)+P O1Uxicq2·sq3−P O0Byi]2+[P O1Uzi·cq1−P O1Uxi·sq2+P O1Uxi·cq2·sq1−P O0Bzi]2

(A2) (liE)2=(‖R O0O2P O2Fi+R O0O1PE−R O0O1P O1Ui‖)2=[P O1Uyi·(cq1·sq3−cq3·sq1·sq2)−P O2Fxi·(sq2·sq4−cq2·cq3·cq4)−P O1Uzi·(sq1·sq3+cq1·cq3·sq2)+lu·(sq1·sq3+cq1·cq3·sq2)+P O2Fxi·(cq2·sq1·sq4+cq3·cq4·sq1−−cq1·cq4·sq3)+P O2Fzi·(cq1·cq2·sq4+cq1·cq3·cq4·sq2+cq4·sq1·sq3)−P O1Uxi·cq2·cq3]2+[P O2Fyi·(cq1·cq3+sq1·sq2·sq3)−P O2Fzi·(cq3·sq1−cq1·sq2·sq3)−P O1Uyi·(cq1·cq3+sq1·sq2·sq3)+ P O1zyi·(cq3·sq1+cq1·sq2·sq3)−lu·(cq3·sq1−cq1·sq2··sq3)+ P O2Fyi·cq2·sq3−P O1Uxi·cq2·sq3]2+[P O1Uxi·sq2−P O2Fxi·(cq2·sq2+cq2·cq3·sq4)+P O2Fyi·(cq2·cq4·sq1+cq3·sq1·sq2·sq4+cq1·sq3·sq4)+P O2Fzi·(cq1·cq2·cq4−cq1·cq3·sq2·sq4−sq1·sq3·sq4)+lu·cq1·cq2−P O1Uzi·cq1·cq2−P O1Uyi·cq2·sq1
